# Transcriptional Silencing by TsrA in the Evolution of Pathogenic Vibrio cholerae Biotypes

**DOI:** 10.1128/mBio.02901-20

**Published:** 2020-11-24

**Authors:** Florence Caro, José A. Caro, Nicole M. Place, John J. Mekalanos

**Affiliations:** a Department of Microbiology and Immunobiology, Harvard Medical School, Boston, Massachusetts, USA; b Department of Biochemistry and Biophysics, Texas A&M University, College Station, Texas, USA; UCLA School of Medicine

**Keywords:** TsrA, *Vibrio cholerae*, horizontal gene transfer, structural modeling, transcriptional regulation

## Abstract

Vibrio cholerae is a globally important pathogen responsible for the severe epidemic diarrheal disease called cholera. The current and ongoing seventh pandemic of cholera is caused by El Tor strains, which have completely replaced the sixth-pandemic classical strains of V. cholerae. To successfully establish infection and disseminate to new victims, V. cholerae relies on key virulence factors encoded on horizontally acquired genetic elements. The expression of these factors relies on the regulatory architecture that coordinates the timely expression of virulence determinants during host infection. Here, we apply transcriptomics and structural modeling to understand how type VI secretion system regulator A (TsrA) affects gene expression in both the classical and El Tor biotypes of V. cholerae. We find that TsrA acts as a negative regulator of V. cholerae virulence genes encoded on horizontally acquired genetic elements. The TsrA regulon comprises genes encoding cholera toxin (CT), the toxin-coregulated pilus (TCP), and the type VI secretion system (T6SS), as well as genes involved in biofilm formation. The majority of the TsrA regulon is carried on horizontally acquired AT-rich genetic islands whose loss or acquisition could be directly ascribed to the differences between the classical and El Tor strains studied. Our modeling predicts that the TsrA protein is a structural homolog of the histone-like nucleoid structuring protein (H-NS) oligomerization domain and is likely capable of forming higher-order superhelical structures, potentially with DNA. These findings describe how TsrA can integrate into the intricate V. cholerae virulence gene expression program, controlling gene expression through transcriptional silencing.

## Abstract (precis)

Pathogenic Vibrio cholerae strains express multiple virulence factors that are encoded by bacteriophage and chromosomal islands. These include cholera toxin and the intestinal colonization pilus called the toxin-coregulated pilus, which are essential for causing severe disease in humans. However, it is presently unclear how the expression of these horizontally acquired accessory virulence genes can be efficiently integrated with preexisting transcriptional programs that are presumably fine-tuned for optimal expression in V. cholerae before its conversion to a human pathogen. Here, we report the role of a transcriptional regulator (TsrA) in silencing horizontally acquired genes encoding important virulence factors. We propose that this factor could be critical to the efficient acquisition of accessory virulence genes by silencing their expression until other signals trigger their transcriptional activation within the host.

## INTRODUCTION

The pathogenic and pandemic potential of the Gram-negative bacterium Vibrio cholerae hinges on three critical properties: the production of cholera toxin (CT), responsible for inducing severe diarrhea in patients; expression of an intestinal colonization factor called the toxin-coregulated pilus (TCP); and possession of either the O1 or the O139 antigen, comprising the O antigen of lipopolysaccharide (1). Two biotypes, classical and El Tor, further define pathogenic V. cholerae O1 strains. Classical strains were responsible for the first six pandemics, while the seventh and ongoing pandemic is caused by El Tor strains (1). CT is encoded by a filamentous phage (CTXΦ) that enters cells using TCP as its receptor, to then integrate into chromosomal sites, converting these bacteria from nontoxigenic to toxigenic (2). The emergence of toxigenic V. cholerae has been postulated to also involve the participation of multiple satellite prophages (3). Given that the genes for TCP biogenesis are located on the horizontally acquired *Vibrio* pathogenicity island 1 (VPI-1) it is clear that the acquisition of multiple accessory genetic elements by V. cholerae allowed its emergence as a human pathogen (3, 4). How the regulation of these foreign genetic elements is fully coordinated by pathogenic strains of V. cholerae remains a mystery.

Several other accessory toxins may play a role in V. cholerae virulence, but the evidence that these are involved in human disease is less clear. For example, the hemolysin HlyA, the multifunctional autoprocessing RTX (MARTX, encoded by *rtxA*) family toxin, and the hemagglutinin/protease HapA have all been proposed to have potential roles in cholera pathogenesis based on results in animal models (5). However, the genes for these toxins are mutated in many V. cholerae clinical isolates: all classical strains carry deletions in *rtxA* and *hlyA*, and most El Tor isolates also carry a deletion in *rtxA* (6–8). Because MARTX and HlyA are proinflammatory, these observations suggest that V. cholerae may be under selective pressure to reduce or eliminate the expression of virulence factors that interfere with the induction of the noninflammatory secretory diarrhea that is most characteristic of cholera (9).

Based on their genome contents and CTXΦ characteristics, the El Tor strains have evolved, and have spread globally in three separate waves, since their emergence in 1961 (4, 10). Wave 1 strains include the reference strain N16961, which was isolated in 1975 in Bangladesh and was the first V. cholerae strain to have its genome sequenced (9, 11). Wave 2 strains constitute an independent lineage between wave 1 and wave 3 strains and were critical in generating wave 3 strains (12). The current wave 3 strains have nearly replaced wave 2 strains and continue to spread globally; wave 3 strains were responsible for the Haiti 2010 cholera epidemic (13). These seventh-pandemic wave 3 “variant strains,” as they are called, have acquired several new properties, including drug resistance encoded by the SXT integrative and conjugative element (ICE) and genetic changes that have altered the expression of several virulence factors.

One such virulence factor is the type VI secretion system (T6SS), a syringe-like nanomachine that injects toxic effector proteins into target cells and kills them (14, 15). The T6SS has been recognized as an important virulence factor in that it can have deleterious effects on both eukaryotic and prokaryotic cells. For example, in a mouse model of cholera, the T6SS was shown to induce intestinal inflammation dependent on VgrG1, an effector with actin cross-linking activity (16), and to facilitate colonization of the host by clearing competing commensal microbiota from its intestinal niche (17).

The pathogenic success of V. cholerae depends not only on acquiring genes that encode critical virulence factors but also on the ability of this organism to sense cues in the host environment and use these to coordinate virulence gene expression at the appropriate time and place. Multiple virulence-regulatory genes have been defined that control the production of CT and TCP in response to numerous chemical signals *in vivo* and *in vitro* (18). Increased cell density of V. cholerae during late stages of intestinal colonization can alter gene expression through quorum sensing and result in the downregulation of virulence traits toward the end of the disease process (19).

Other regulators also negatively influence virulence gene expression. For example, the type VI secretion system regulator A (TsrA, VC0070) reduces the expression of T6SS, TCP, CT, and their regulator ToxT and increases the apparent proteolytic activity of the HapA protease (20). Increases in virulence factor expression, as seen in the Δ*tsrA* mutant, allow this strain to colonize the intestines of neonatal mice significantly better than an isogenic wild-type strain (20).

TsrA has weak amino acid sequence homology to the histone-like nucleoid structuring protein (H-NS) (20), a highly abundant and ubiquitous protein found in Gram-negative bacteria that functions as a transcriptional repressor of AT-rich and DNA regions with planar curvature commonly found at bacterial promoters (21–23). H-NS molecules oligomerize to form a superhelix that interacts with DNA and acts on its topology, affecting DNA supercoiling and condensation (24). Both in Escherichia coli and in Salmonella enterica serovar Typhimurium, H-NS has been found to selectively silence the transcription of genes present on horizontally acquired genetic islands, including those encoding major virulence functions (21–23). In V. cholerae, H-NS regulates the expression of ∼20% of genes with functions associated with virulence, surface attachment, biofilm formation, motility, and chemotaxis (25). V. cholerae Δ*h-ns* mutants display increased cholera toxin expression, reduced flagellar motility, and reduced colonization efficiency (26, 27). It is not known if TsrA, as an H-NS ortholog, controls expression by a similar mechanism that is consistent with transcriptional silencing of genes based on their high AT abundance.

To expand our knowledge of TsrA structure and function, and its role in the evolution of V. cholerae pandemic strains, we applied transcriptomics and protein structure homology modeling of TsrA in multiple V. cholerae isolates representing strains from different eras of the bacterium’s evolution. Our data establish TsrA as a transcriptional silencer of major virulence genes, including CT, TCP, and T6SS, as well as genes encoding functions involved in biofilm formation. The vast majority of TsrA regulon members are localized within horizontally acquired genetic islands, suggesting that TsrA has a role in “xenogeneic silencing” similar to that described for H-NS (21). The TsrA regulon is relatively conserved among classical, El Tor wave 1, and wave 3 V. cholerae strains; the presence or absence of genetic islands in these strains is the main factor contributing to the differences observed between them. Our structure homology modeling demonstrates that TsrA shares high similarity with the H-NS oligomerization domain. Our modeling also suggests that TsrA forms a higher-order superhelix, albeit of different proportions from those observed with H-NS, which may drive its binding specificity.

## RESULTS

### TsrA silences *V. cholerae* virulence genes located on accessory genetic islands.

To identify the role of TsrA as a transcriptional regulator of virulence over the course of V. cholerae evolution, we chose three strains, one representative of the sixth pandemic and two representative of the seventh pandemic. They are the sixth-pandemic classical Ogawa 395 (O395) clinical isolate from India (1965), O395-N1 (28); the wave 1 El Tor strain C6706, a clinical isolate from the 1991 outbreak in Peru (29); and the wave 3 El Tor strain Haiti H1, a clinical isolate from the 2010 outbreak in Haiti (30). The O395 derivative O395-N1 is a vaccine strain with an internal deletion in *ctxA* that renders the toxin inactive, and thus, the strain is nontoxigenic and a relatively low biohazard risk for a classical strain, given that this biotype is extinct as a natural epidemiological threat (28). The V. cholerae quorum-sensing pathway has been documented to accumulate spontaneous inactivating mutations, particularly in the two key regulators LuxO and HapR (31, 32). The C6706 strain used in this study is a streptomycin-resistant laboratory derivative of the original clinical isolate that acquired a spontaneous luxO* mutation (G333S), which we repaired to its original wild-type (WT) allele. The Haiti H1 clone used is the original clinical isolate (30).

**FIG 1:**
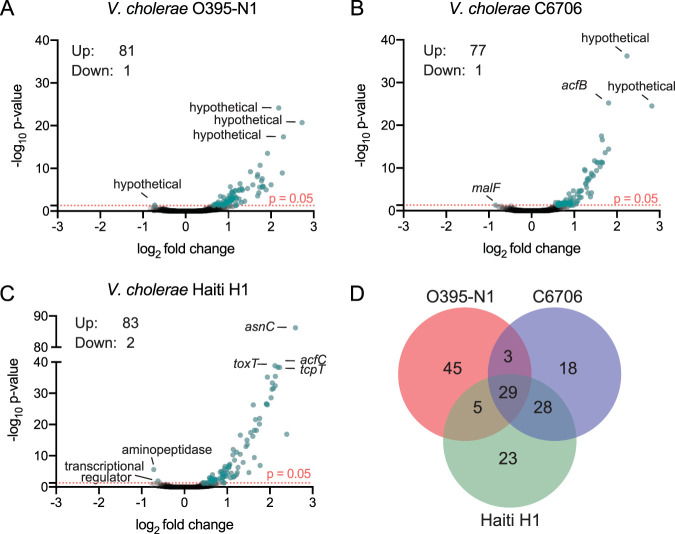
TsrA is a transcriptional repressor of a relatively small set of V. cholerae genes. (A to C) Volcano plot representations of differential expression analysis of the wild type versus the isogenic Δ*tsrA* mutant for V. cholerae O395-N1 (A), C6706 (B), and Haiti H1 (C) RNA-seq data sets. The *x* axis shows the log_2_ fold change in expression, and the *y* axis shows the log odds of a gene being differentially expressed (ac, –log_10_ *P* value). Genes are shown in blue if they pass the absolute confidence (ac) threshold of ≥1.3 (equivalent to a *P* value of ≤0.05, pink dotted line). The identities of the top three genes with the highest ac values, and the few genes with negative fold changes, are given. (D) Venn diagram of the number of genes that are members of the TsrA regulon in each of the strains.

We performed transcriptome sequencing (RNA-seq) analysis of three biological replicates of either the WT or an isogenic *ΔtsrA* mutant of each strain during exponential growth in liquid culture (see Table S1 in the supplemental material). Differentially expressed genes were determined using the DEseq2 software pipeline (33), and genes with an absolute confidence (ac) value of ≥1.3 (or a *P* value of ≤0.05) are considered to be differentially expressed. Only ∼80 genes (2% of the V. cholerae genome) were differentially regulated in the *ΔtsrA* mutants of the three strains profiled; the majority of genes displayed an increase in mRNA levels in the mutant strain, indicating that TsrA acts primarily as a transcriptional repressor in V. cholerae (Fig. 1A and Table S2). Transcripts from only one or two genes in each data set were present in significantly lower abundance in the Δ*tsrA* mutants. These encode a hypothetical protein (VC0395_RS21005) in O395-N1, the maltose transporter membrane protein MalF (VCA0944) in C6706, and an AraC-family regulator (N900_RS05235) and an X-Pro aminopeptidase (N900_RS00355) in Haiti H1. In contrast to previous findings, our data showed no effect of Δ*tsrA* on *hapA* expression (20). Given that HapA activation was measured in the earlier study as a function of protease activity, it is possible that the *tsrA* mutant affects this protein’s activity at the posttranscriptional level. Also, the C6706 strain used by Zheng et al. (20) may contain the luxO* allele, genetically restored to its wild-type form in this study, which may affect *hapA* expression in the *tsrA* mutant. Alternatively, since *hapA* expression is controlled by RpoS and is activated only in stationary phase, the effects of TsrA on *hapA* may not be observable in mid-log-phase cultures, such as those used in this study.

10.1128/mBio.02901-20.1 TABLE S1 Illumina sequencing statistics. The number of high-quality reads (pass filter reads) obtained by Illumina sequencing, the number of reads mapped to each of the two chromosomes, and the percentage of reads mapped are listed for each of the strains analyzed in this study. Download Table S1, XLSX file, 0.01 MB. Copyright © 2020 Caro et al. 2020 Caro et al. This content is distributed under the terms of the Creative Commons Attribution 4.0 International license.

10.1128/mBio.02901-20.2 TABLE S2 TsrA RNA-seq and differential expression data sets. The first sheet explains the column values used. RNA-seq data obtained for strains O395-N1, C6706, and Haiti H1 and their isogenic Δ*tsrA* mutants are listed on separate sheets labeled with the corresponding strain name. The differentially expressed subset of genes is listed on sheets labeled with the strain name followed by DiffE. Download Table S2, XLSX file, 1.0 MB. Copyright © 2020 Caro et al. 2020 Caro et al. This content is distributed under the terms of the Creative Commons Attribution 4.0 International license.

**FIG 2:**
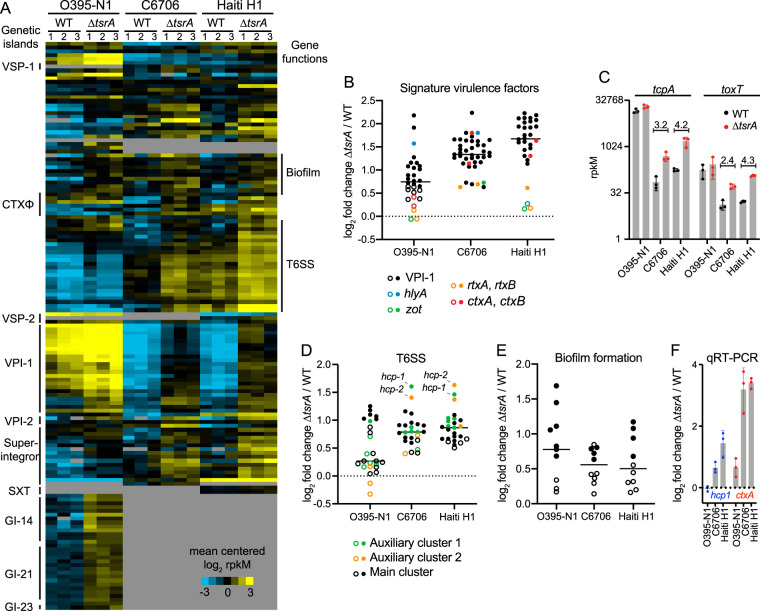
The majority of genes in the TsrA regulon are encoded on genetic islands that are most highly activated in the Haiti H1 Δ*tsrA* strain. (A) Heat map of mean-centered log_2_-transformed expression values of members of the TsrA regulon in each of the three biological replicates of each wild-type and mutant strain. Each row in the heat map corresponds to a gene, and each column in the heat map corresponds to a sample (identified above the heat map); if no ortholog of the gene is found in the strain, the field is shaded gray. Genetic islands are indicated on the left of the heat map, and clusters of biofilm formation and T6SS genes are indicated on the right. (B, D, and E) Fold changes from the wild type in the expression of genes encoding signature virulence factors (B), the T6SS (D), and biofilm formation functions (E) in the Δ*tsrA* mutant. Filled circles indicate genes considered significantly differentially expressed and part of the TsrA regulon in the corresponding strain (ac, ≥1.3). Open circles indicate genes not regulated by TsrA. Horizontal bars indicate mean values. (C) *tcpA* and *toxT* mRNA abundances expressed in reads per kilobase per million (rpkM) (*y* axis on a log_2_ scale) in either the WT (black circles) or the Δ*tsrA* mutant (red circles) of each of the three strains analyzed. Numbers above the C6706 and Haiti H1 data sets indicate the fold rpkM change between the WT and the Δ*tsrA* mutant. Error bars indicate standard deviations of rpkM measurements for the three independent biological replicates. (F) qRT-PCR measuring the fold change in *hcp1* (blue) and *ctxA* (red) mRNA abundances between the WT and the Δ*tsrA* mutant in each of the strains analyzed. Error bars represent the standard deviations of measurements obtained from three independent biological replicates.

To compare the transcriptional profiles of the genes in the TsrA regulon in each of the strains, we created a list of orthologous genes using blastn reciprocal best-hits analysis (Fig. 2A and Table S3) (34). The TsrA regulons of the El Tor strains largely overlapped; ∼70% of genes’ mRNA levels were elevated in the Δ*tsrA* mutants of both C6706 and Haiti H1, compared to only a ∼40% overlap between the classical O395-N1 strain and the El Tor strains. Of the 45 genes unique to the O395-N1 TsrA regulon, 30 are encoded on genetic islands GI-14, GI-21, and GI-23, which are absent in the El Tor strains. In general, the vast majority of genes regulated by TsrA are encoded on horizontally acquired genetic islands, including the superintegron present on chromosome 2, VPI-1, VPI-2, CTXΦ, VSP-1 and VSP-2 (present only in the two El Tor strains), and SXT (present only in Haiti H1). The three strains have 29 differentially regulated genes in common, including members of the VPI-1 and VPI-2 genetic islands, the T6SS gene clusters, and genes encoding functions involved in biofilm formation (Fig. 1B, D, and E). Gene clusters encoding functions involved in biofilm formation do not appear to be the result of horizontal gene transfer events, but they are, nonetheless, part of the TsrA regulon. In contrast, T6SS gene clusters may have been acquired by horizontal gene transfer, since they have been found encoded on excisable mobile genetic elements (35), and transfer of T6SS effector-immunity pairs between V. cholerae strains has been experimentally demonstrated (36). Counting the T6SS gene clusters, around 80% of the TsrA regulon members in all three strains are encoded on mobile genetic elements, implicating TsrA in the silencing of horizontally acquired V. cholerae genes (Table 1).

**TABLE 1:** Numbers of genes repressed by TsrA that are present on genetic islands

Genetic island	No. of genes repressed by TsrA in strain:		
	O395-N1	C6706	Haiti H1
CTXΦ	0	5	3
Superintegron	4	10	11
O395 GI14	13	–	–
O395 GI21	16	–	–
O395 GI23	1	–	–
SXT	–	–	2
VPI-1	17	24	24
VPI-2	2	3	2
VSP-1	–	1	1
VSP-2	–	0	2
T6SS	7	18	21
Total no. of genes (% of regulon)	60 (73.2)	61 (78.2)	66 (77.6)

10.1128/mBio.02901-20.3 TABLE S3 RNA-seq and differential expression analysis of orthologous genes. This table lists expression values, fold change, and absolute confidence of the intersect between all TsrA regulon members of each of the three strains analyzed in this study. Also described is the genetic island each gene belongs to, as well as the position of each gene in the Fig. 2A heat map. Download Table S3, XLSX file, 0.05 MB. Copyright © 2020 Caro et al. 2020 Caro et al. This content is distributed under the terms of the Creative Commons Attribution 4.0 International license.

The TsrA regulons of both El Tor strains encompassed larger sets of VPI-1 genes (24 genes) than the classical strain (17 genes) (Table 1). The fold activation measured in the Δ*tsrA* mutant was also progressively higher on average than in the classical strain (2.1-, 2.5-, and 3.5-fold for O395-N1, C6706, and Haiti H1, respectively [Fig. 2B]). For example, *tcpA* and *toxT* absolute transcript abundances were much higher in the WT classical strain than in the El Tor strains but were not significantly affected in the Δ*tsrA* mutant, and thus, these genes are not part of the TsrA regulon in this strain. Their mRNA levels, however, increased significantly in the Δ*tsrA* mutants of both El Tor strains, and the fold increase was larger in Haiti H1 than in C6706 (Fig. 2C). Conversely, transcripts encoding functions involved in biofilm formation were, on average, more abundant in the O395-N1 Δ*tsrA* mutant than in El Tor Δ*tsrA* mutants, and *vpsS* was the most abundant transcript in the former (Fig. 2E). VpsS is a hybrid histidine kinase whose activity increases biofilm formation through the quorum-sensing pathway and is repressed by TsrA only in O395-N1 (56). In comparison, in Haiti H1, TsrA influences biofilm formation not by repressing *vpsS* but instead by repressing *vpsT*, a major activator of biofilm formation genes that requires binding to c-di-GMP in order to associate with DNA and regulate transcription (37). Thus, in both strains, TsrA negatively affects the expression of genes involved in biofilm formation but does so by acting on two different activators of the circuit that sense distinct environmental factors.

The TsrA regulon included a more complete set of 18 or 21 T6SS genes for C6706 or Haiti H1, respectively, compared to the classical strain set of 7 (Fig. 2D; Table S3). On average, transcripts encoding T6SS functions were also more abundant in both El Tor Δ*tsrA* mutant strains than in the classical strains; *hcp-1* and *hcp-2* were the most abundant transcripts (Fig. 2D and F; Table S3). The data show that in O395-N1, only one gene encoded on T6SS auxiliary cluster 1 and the main T6SS cluster genes are regulated by TsrA. It is worth noting that while *vipA* is among the T6SS transcripts displaying significantly increased abundance in the O395-N1 Δ*tsrA* mutant, this gene contains a documented frameshift mutation that results in a truncated protein product (38). Given the essential structural role that VipA plays in T6SS sheath assembly, this mutation likely renders the T6SS apparatus inactive in classical strains (15). Thus, while the transcriptional wiring of T6SS expression through TsrA remains partially conserved between classical and El Tor strains, the phenotypic outcome is vastly different, with El Tor strains experiencing a greater increase of a larger set of T6SS components that assemble into a functional apparatus.

We next focused on the role of TsrA in the regulation of V. cholerae toxins. Transcripts of *ctxA* and *ctxB*, encoding both subunits of cholera toxin, were elevated in the Δ*tsrA* mutants of both El Tor strains, but not in the classical strain (Fig. 2A, B, and F). Transcript levels of other genes on the CTXΦ phage were also elevated in the C6707 Δ*tsrA* mutant. A toxin-related locus adjacent to the CTXΦ phage integration site in El Tor strains was also transcriptionally upregulated, despite the fact that this locus appears to contain a pseudogene in both biotypes. Specifically, *rtxA* contains a naturally occurring 7-kb deletion and a premature stop codon in O395-N1 and in Haiti H1, respectively, that renders the toxin nonfunctional in these strains (6, 7). Because the contribution of MARTX to virulence has been found to be redundant with that of HlyA in an adult mouse model (39), we analyzed the expression of this gene in the Δ*tsrA* mutant and found that it is highly abundant in O395-N1 and C6706 but not in Haiti H1. However, O395-N1 *hlyA* also contains a premature stop codon generating an inactive toxin (8). Thus, even under conditions of elevated *hlyA* transcript levels in a Δ*tsrA* mutant, expression of the truncated protein would not compensate for the loss of MARTX in classical strains.

Collectively, our data show that TsrA silences many V. cholerae virulence genes that are located predominantly on accessory genetic islands or prophages. In the absence of TsrA, the expression of these accessory virulence genes increases progressively from the much older classical isolates to the more recent El Tor wave 1 strains to wave 3 strains. These data suggest that TsrA is another mechanism (in addition to H-NS) for silencing recently acquired genes until the recipient strain can full adapt to their presence and expression in a new selective niche, such as the human host (21). Additionally, while TsrA-dependent changes in the V. cholerae transcriptome are relatively conserved across strains, the phenotypic consequences of this regulation in many instances will be determined by whether the transcribed gene encodes a functional protein or not.

### Structural homology modeling predicts a TsrA superhelix scaffold.

TsrA is highly conserved within Vibrionales and 100% identical at both the nucleotide and amino acid levels in the three strains analyzed here. Outside this taxon, blastp searches identify TsrA only in the gammaproteobacterium *Plesiomonas* sp., a genus represented by a single species, Plesiomonas shigelloides, and show that it shares 49% amino acid identity with its V. cholerae counterpart. Homology and structure prediction analysis using HHPRED showed that TsrA bears a weak similarity to the N-terminal oligomerization domain of the heat-stable nucleoid structuring protein (H-NS) of Escherichia coli (20, 34, 40). H-NS is a small, abundant protein found in Gram-negative bacteria that preferentially binds to AT-rich DNA with planar curvature and mediates chromosomal DNA condensation (23). Both in Escherichia coli and in *Salmonella* serovar Typhimurium, H-NS was found to selectively silence the transcription of genes present on horizontally acquired genetic islands, including those encoding major virulence functions (21–23). V. cholerae H-NS regulates the expression of ∼18% of genes with functions including virulence, surface attachment, biofilm formation, motility, and chemotaxis (25, 41).

**FIG 3:**
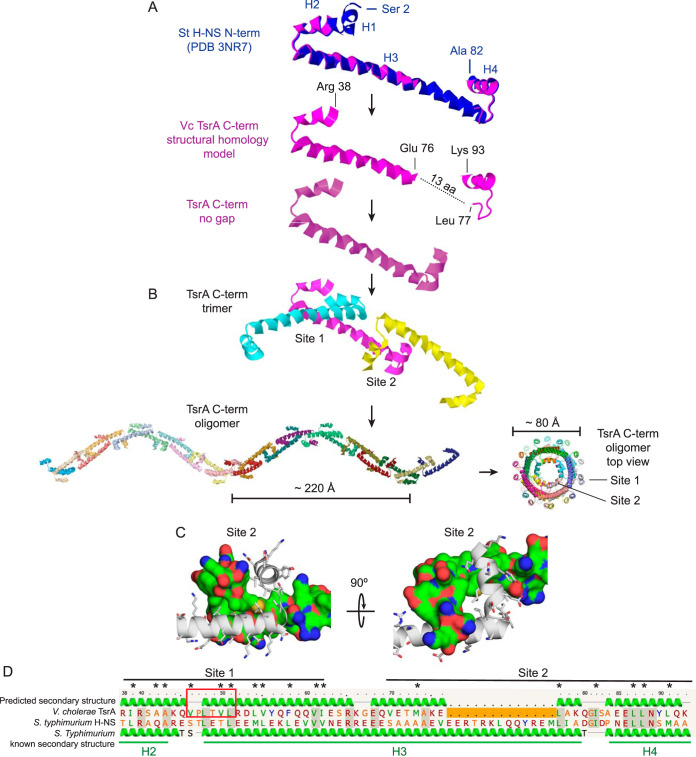
TsrA structural homology modeling. (A) The V. cholerae N terminus of TsrA (Arg 38 to Lys 93) (magenta) is structurally homologous to the N-terminal *S.* Typhimurium H-NS oligomerization domain (Ser 2 to Ala 82) (blue) but contains a gap (dotted line) that predicts a shorter protein fold than that of H-NS (by 13 amino acids [aa] or ∼18 Å). The TsrA N terminus (residues 1 to 37) is excluded from the modeled structure, thus omitting alpha helix H1. The known alpha helices of *S.* Typhimurium, H1 to H4, are indicated. (B) TsrA oligomerization sites 1 and 2 were modeled after the structure of sites 1 and 2 in *S.* Typhimurium H-NS to build a TsrA trimer and a superhelix, with a ∼220 Å rise and a ∼70 Å diameter. Site 1 is always positioned on the exterior surface of the helix, while site 2 always points inward to the center of the helix. (C) Detailed view of the TsrA site 2 model structure, where hydrophobic interactions of conserved residues are analogous to those observed in *S.* Typhimurium H-NS oligomerization site 2. One protomer is displayed as a secondary structure and stick model; the other, as molecular surface. The surface is colored to highlight different residue properties: blue, nitrogen atoms; red, oxygen atoms; green, hydrophobic atoms; yellow, sulfur atoms. (D) Amino acid sequence alignments of the modeled domains of V. cholerae TsrA and *S.* Typhimurium H-NS. Predicted and known secondary structures are shown above and below for TsrA and H-NS, respectively, and TsrA residue numbers are shown for reference. Residues that compose sites 1 and 2 are marked with a black line. Alpha helices H2, H3, and H4, derived from the *S.* Typhimurium H-NS structure, are underlined in green. The TsrA site 1 proline box motif HPXXHH that caps the alpha helix, where H is hydrophobic, and X is any amino acid, is boxed in red. Conserved hydrophobic residues that support the site 2 structure in panel C are marked with asterisks.

Because V. cholerae TsrA targets genes found predominantly on genetic islands and displays some amino acid sequence homology to H-NS, we hypothesized that these two proteins share functional and structural similarities. To test this, we analyzed TsrA structure using the protein homology/analogy recognition engine (Phyre) (42) and found high structural overlap (96.4% confidence) of the TsrA C-terminal domain (residues 38 to 93) with the N-terminal oligomerization domain of *S.* Typhimurium H-NS (PDB code 3NR7) (24). The N-terminal oligomerization domain (residues 1 to 83) of H-NS is connected by a flexible linker to a C-terminal DNA-binding domain which is not included in the *S.* Typhimurium structure. *S.* Typhimurium H-NS protomers interact “head-to-head” via two short N-terminal helices, H1 and H2 (site 1), and “tail-to-tail” via alpha-helices H3 and H4 (site 2) (24). Together, a chain of H-NS protomers form a superhelix that is predicted to interact with DNA, affecting DNA supercoiling and condensation (43). The modeled TsrA structure predicts both sites 1 and 2 to exist but in closer proximity (by precisely 13 amino acids) than in *S.* Typhimurium H-NS (Fig. 3A). Guided by the site 1 and 2 interfaces of the *S.* Typhimurium H-NS structure, we created a model for a chain of TsrA molecules that form a higher-order structure analogous to that proposed for H-NS (35). In this context, the C termini of two TsrA protomers interlock “tail-to-tail” at site 2 in an antiparallel fashion and are stabilized by a hydrophobic core (formed by conserved residues Leu 88, Leu 87, Ile 82, Leu 77, Met 73, Val 70, and Leu 91) (Fig. 3C). The modeled TsrA structure excludes the N terminus of the protein (residues 1 to 37) but includes one of the two alpha-helices (H2) that shape the oligomerization domain’s site 1. The short H2 helix is followed by H3, which in TsrA is capped by a proline box motif that stabilizes the N terminus of this helix (Fig. 3D) (44). The modeled TsrA superhelix is shorter, ∼220 Å in pitch, and has a narrower diameter (∼70 Å) than the H-NS superhelix (Fig. 3B). Notably, while site 2 is positioned always on the inside of the helix, site 1 always faces the helix’ outer surface, presumably available to interact with DNA. Indeed, the curved distance between two sites 1 is ∼70 Å, making it capable of fitting twice the pitch of B-DNA (two times 34 Å) (45). These structure predictions suggest that TsrA could function as a superhelical protein scaffold, similar to H-NS, that interacts with extensive stretches of DNA rather than as a classical transcription factor interacting with shorter, specific DNA sequence motifs.

**FIG 4:**
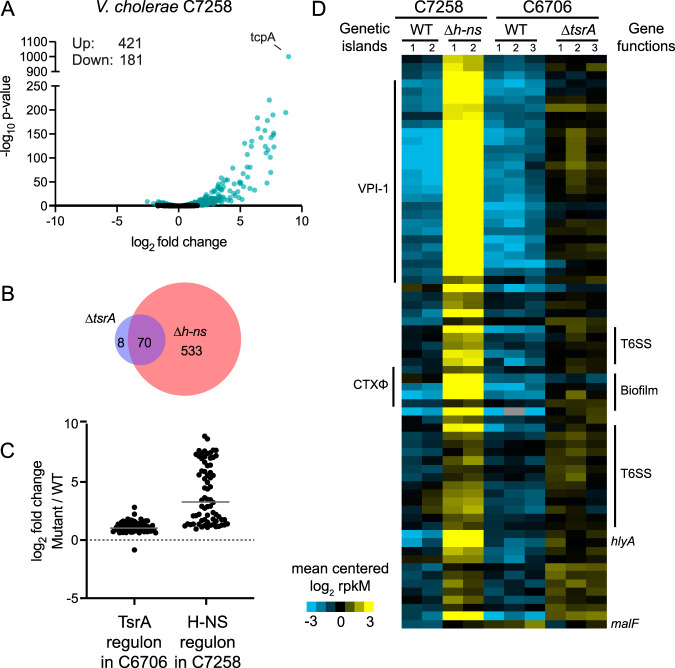
The V. cholerae TsrA and H-NS regulons largely overlap. (A) Volcano plot representation of the differential expression analysis of the wild type versus the isogenic Δ*h-ns* mutant of V. cholerae C7258 RNA-seq data sets. The *x* axis shows the log_2_ fold change in expression, and the *y* axis shows the log odds of a gene being differentially expressed (ac, –log_10_ *P* value). Genes are shown in blue if they pass the absolute confidence threshold (ac, ≥1.3; equivalent to a *P* value of ≤0.05). (B) Venn diagram of the overlap between the TsrA and H-NS regulon members of V. cholerae strains C6706 and C7258, respectively. (C) Fold changes between the Δ*tsrA* and Δ*h-ns* mutants and wild-type strains of C6706 and C7258 in the expression of all differentially regulated genes in either regulon. The gray horizontal bar represents the mean fold change in each regulon, 2.2- and 16-fold, for the TsrA and H-NS regulons, respectively. (D) Heat map of mean-centered, log_2_-transformed expression values of members of the H-NS and TsrA regulons. Two and three biological replicates are depicted for the H-NS (47) and TsrA (this study) data sets, respectively. Each row in the heat map represents a gene, and each column in the heat map represents a sample (identified above the heat map); if no ortholog of the gene is found in the strain, the field is shaded gray. Genetic islands are indicated on the left of the heat map, and clusters of biofilm formation and T6SS genes are indicated on the right.

To determine whether the structural similarities between H-NS (VC1130) and TsrA could be extended to functional similarities, since both are transcriptional regulators of V. cholerae gene expression, we analyzed a previously published RNA-seq data set for strain C7258, a wave 1 El Tor clinical isolate from the 1991 Peru epidemic and its isogenic Δ*h-ns* mutant, obtained under the same growth conditions as our own C6706 data set for the Δ*tsrA* mutant and its parental strain (Fig. 4A; Table S4) (25, 46, 47). While the H-NS regulon includes a larger set of genes than the TsrA regulon (603 versus 78), they largely overlap, and 70 of the 78 genes whose transcripts were in higher abundance in the Δ*tsrA* mutant were found similarly elevated in the Δ*h-ns* mutant (Fig. 4B and D). One exception is *malF*, the only transcript whose levels were found to decrease in the Δ*tsrA* mutant but to increase in the Δ*h-ns* mutant. The increase in mRNAs shared by the TsrA and H-NS regulons tends to be, on average, larger in the absence of H-NS than in the absence of TsrA (16-fold and 2.2-fold, respectively) (Fig. 4C and D). For example, *tcpA* mRNA levels in the Δ*h-ns* mutant are 488-fold higher than in the WT C7258, whereas the largest fold increase measured between WT C6706 and its Δ*tsrA* mutant is only 7-fold for the hypothetical protein VCA0874. In contrast to H-NS, which positively influences roughly one-third of the genes in its regulon, TsrA acts predominantly as a negative control element. However, as has been suggested previously, it is possible that positive regulation of gene expression by H-NS occurs by indirect or posttranscriptional mechanisms (48). H-NS represses the full extent of the operon that encodes proteins that generate the *Vibrio* polysaccharide as well as the operon that encodes the major biofilm proteins; these include the *Vibrio* polysaccharide 1 (*vps-1*), rugosity and biofilm structure modulator (*rbm*), and *vps-2* gene clusters. TsrA, on the other hand, regulates only four genes present in this cluster in strain C6706. Thus, TsrA and H-NS exert largely overlapping functions as negative regulators of the majority of the members of the TsrA regulon.

10.1128/mBio.02901-20.4 TABLE S4 H-NS RNA-seq and differential expression data sets. Shown is RNA-seq analysis of the V. cholerae C7258 clinical isolate from a 1991 Peru outbreak, serogroup O1 Ogawa, El Tor biotype, by Ayala et al. (47). The first sheet explains the column values used. The subsequent sheets list the RNA-seq and differential expression analysis as well as the list of genes that intersect between the two data sets. The last data set lists the genes in the order of the heat map depicted in Fig. 4D. Download Table S4, XLSX file, 0.4 MB. Copyright © 2020 Caro et al. 2020 Caro et al. This content is distributed under the terms of the Creative Commons Attribution 4.0 International license.

**FIG 5:**
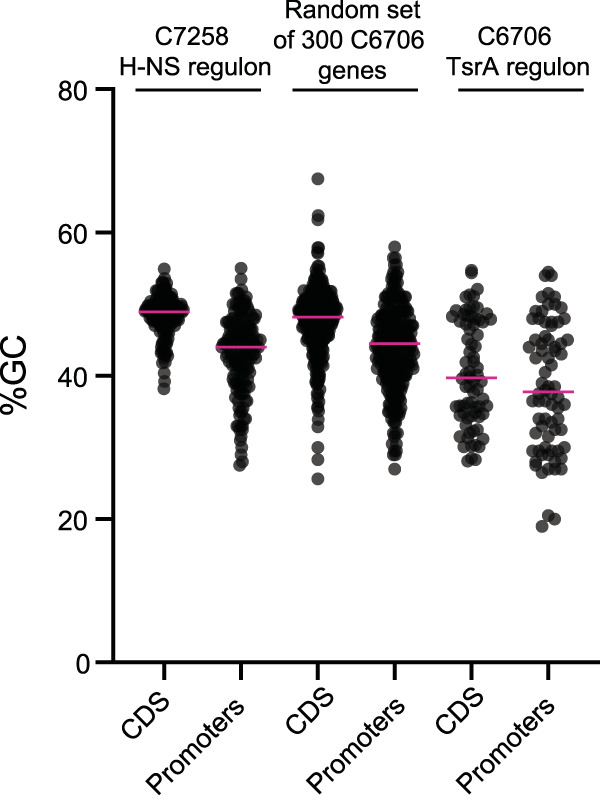
The DNA sequences of TsrA regulon members are AT rich. Shown are the GC percentages of either the coding sequences (CDS) or the 200-nucleotide upstream regions of members of the C7258 H-NS regulon (including only those genes negatively regulated by H-NS), the C6706 TsrA regulon, or a randomly selected set of 300 C6706 genes. Pink horizontal lines represent the mean of each distribution.

Because of the functional and structural similarities found between TsrA and H-NS, we hypothesized that TsrA may also display a preference for regulating AT-rich DNA. To determine this, we analyzed the GC content of every C6706 TsrA regulon member and compared that to either negatively regulated H-NS regulon members or a set of randomly selected genes. For each set of genes analyzed, the GC percentage of either the coding sequence or a 200-nucleotide sequence immediately upstream of the ATG, as a proxy for a promoter, was determined. Among the three sets of genes analyzed, TsrA regulon members displayed the lowest GC content: 40.5% compared to 48.1% and 47.5% for H-NS and the random set of genes, respectively (Fig. 5). In the case of the proxy set of promoter regions, the GC percentages were 38.5%, 43.2%, and 43.8% for the TsrA genes, H-NS genes, and the random set of genes, respectively. These data indicate that like H-NS, TsrA exerts its regulatory function preferentially on regions of DNA with an AT-enriched nucleotide content.

## DISCUSSION

Here, we show that V. cholerae TsrA negatively affects the mRNA levels of a small but significant fraction of genes encoding major virulence factors of V. cholerae, including CT, TCP, accessory toxins, and the T6SS. We found that in the absence of TsrA, the fold increase in the transcript abundance of many of these signature virulence factors becomes progressively larger from classical strains to El Tor wave 1 strains to El Tor wave 3 strains. This is the case, for example, for *tcpA* and *toxT*, for which mRNA levels are most elevated in the Haiti H1 strain relative to C6706 and are not part of the classical O395-N1 TsrA regulon. Wave 3 El Tor strains, including Haiti H1, have been shown to display a more virulent phenotype than the prototypical wave 1 El Tor strains such as C6706, a trait attributed to the increased production of CTx and hemolysin and a reduced ability to form biofilms (49). In agreement with this notion, our data suggest that a Haiti H1 Δ*tsrA* mutant is likely to display a hypervirulent phenotype *in vivo* similar to, if not more severe than, the one observed for the C6706 Δ*tsrA* mutant (20). Our data also add TsrA to the complex network of transcriptional regulators of biofilm formation. The majority of the genes in the TsrA regulon are found encoded on genetic islands. Indeed, genetic islands are the major feature differentiating TsrA regulons in classical, El Tor wave 1, and El Tor wave 3 strains. For example, in the classical O395-N1 strain, TsrA silences the expression of GI-14, GI-21, and GI-23, genetic islands that are absent in the El Tor strains. Conversely, TsrA silences genes found on the SXT element found in Haiti H1 and all variant strains of wave 3 of the seventh pandemic. This integrative and conjugative element encodes resistance to several antibiotics, and its first acquisition lies at the point of transition from wave 1 to wave 2 (10). This implicates TsrA in the repression of both older genetic islands and newly acquired genetic elements crucial for the epidemic success of current pandemic V. cholerae strains.

H-NS has been hypothesized to serve a role in silencing horizontally acquired genes, keeping them in an inert state to limit the fitness cost to the cell until they are incorporated into its regulatory architecture (23). The tight overlap between the TsrA and H-NS regulons suggests that TsrA acts as an additional gatekeeper of gene expression, particularly for those genes encoding functions of consequence to V. cholerae pathogenesis. Our structural model predicts that TsrA is capable of oligomerizing via its C-terminal domain in a fashion analogous to that of H-NS but yielding a superhelix with different physical proportions. In both cases, H-NS and TsrA molecules form a chain of head-to-head and tail-to-tail linked molecules, but in the case of TsrA, each link in the chain is shorter, resulting in a superhelix with a shorter pitch and a narrower diameter. H-NS has been shown to repress a much larger set of genes than TsrA. This apparent promiscuity probably arises from the fact that H-NS does not bind to a consensus sequence but rather to a curved DNA structure commonly found in promoter regions (21). The downsizing of the TsrA superhelix may restrict its DNA binding capacity to fewer regions in the genome with a specific topology, which could explain its limited effect on gene expression. The N-terminal domain of TsrA does not contain homology to any known DNA-binding domains, raising the question of whether this protein is capable of interacting directly with DNA or not. TsrA may interact with other proteins that act as bridges to DNA. While the crystal structure of TsrA remains to be determined, the high structural homology between the TsrA and H-NS oligomerization sites raises the intriguing possibility of these two proteins forming heteromeric structures. In this scenario, a TsrA–H-NS heteromeric superhelix would make DNA contacts via the H-NS DNA-binding domain. Depending on the ratio of either of these proteins, the assembled superhelix could adopt different shapes, exerting a range of effects on DNA topology and consequently gene expression. Indeed, H-NS has been proposed to interact with related but distinct proteins (48). Such an interplay between these two proteins could explain how they both influence the expression of a common set of target genes, whereby only the combined action of both proteins results in full repression. Supporting this notion is the fact that TsrA regulon members’ DNA sequence is enriched in AT, a feature also shared with H-NS. It is presently unclear what chemical or physical cues TsrA senses to relieve repression. Given their functional and structural overlap, the mechanisms for TsrA derepression may be analogous to those described for H-NS, which include displacement by transcriptional activators, increase in temperature, and osmolyte and pH shifts (48, 50, 51). Interestingly, unlike H-NS, which is widespread in Gram-negative bacteria, TsrA can be found outside *Vibrionales* in only one other species of bacteria, P. shigelloides. These bacteria were formerly classified within the *Vibrionaceae* but, as a result of biochemical and genetic analysis, are now classified as *Enterobacterales*. It is hard to speculate where TsrA arose first, whether in *Plesiomonas* or vibrios, but its restricted phyletic distribution suggests that TsrA is unlikely to evolve via horizontal gene transfer.

In summary, the convergence of our transcriptomic and structural modeling data shows that TsrA structure and function resemble those of H-NS. Both proteins negatively regulate virulence genes often encoded on genomic elements that are not part of the core genome. Our data suggest that TsrA modulates V. cholerae virulence by focusing regulation on accessory genetic elements (phage and chromosomal islands) that encode virulence factors needed for successful human host colonization and dissemination. V. cholerae evolution from classical to El Tor wave 1 and 3 strains involved loss or acquisition of many horizontally transferred genetic islands. TsrA repression of these genetic islands and phage elements may represent a mechanism to curtail fitness loss until these elements are integrated into the regulatory architecture of the cell. A deeper understanding of how TsrA and H-NS collaborate or act independently to modulate gene expression in recently emerged pathogens such as V. cholerae El Tor strains of the first and third waves will provide greater insights into how V. cholerae evolved from a successful geographically confined human pathogen to a pandemic threat.

## MATERIALS AND METHODS

### Bacterial strains, plasmids, and culture conditions.

The strains, plasmids, and primers used in this study are listed in Table S5 in the supplemental material. V. cholerae strains O395-N1 (28), C6706 (29), and Haiti H1 (52) were used as parental strains in this study. E. coli Sm10 λpir was used as the host strain for cloning and conjugation plasmids. To create the *tsrA* (VC0070) in-frame clean deletion mutant, ∼500-bp gene-flanking regions were PCR amplified using C6706 genomic DNA as the template with primer oFC729-32 and were cloned into pWM91 (plasmid pFC42), which was used for subsequent in-frame allelic exchange of the gene, as described elsewhere (53). To repair luxO* in C6706, a ∼500-bp fragment was amplified from genomic DNA isolated from a C6706 laboratory clone with a wild-type *luxO* (VC1021) allele and was cloned into PWM91 (plasmid pFC39) for allelic exchange as described elsewhere (57). Cloning of allelic exchange plasmids was performed using the NEBuilder HiFi DNA Assembly cloning kit (New England Biolabs). The Δ*tsrA* mutant and *luxO* alleles were confirmed by PCR and sequencing using primers oFC58-61 and oFC062-5, respectively. Bacteria were routinely grown aerobically at 37°C in LB Lennox (1% tryptone, 0.5% yeast extract, 0.5% NaCl) in broth or on LB agar plates. Antibiotics were added to the medium at the following concentrations: 100 μg/ml streptomycin, 20 μg/ml chloramphenicol (E. coli), 0.5 mg ml^−1^ chloramphenicol (V. cholerae), and 100 μg/ml carbenicillin.

10.1128/mBio.02901-20.5 TABLE S5 Strains, plasmids, and primers used in this study. Download Table S5, XLSX file, 0.01 MB. Copyright © 2020 Caro et al. 2020 Caro et al. This content is distributed under the terms of the Creative Commons Attribution 4.0 International license.

### RNA preparation and RNA-seq.

For each of the three biological replicates, the WT and isogenic Δ*tsrA* mutant strains were streaked out from glycerol stocks onto LB agar plates and incubated overnight at 37ºC. The next day, one colony was inoculated into a beveled flask containing 25 ml LB and was grown at 37ºC in an orbital shaker at 300 rpm until the culture reached an optical density at 600 nm [OD_600_] of 0.5. Bacteria were harvested by centrifugation, resuspended in 1 ml of TRIzol (Thermo Fisher Scientific), and incubated for 5 min at room temperature before the addition of 200 μl of chloroform. Samples were centrifuged at a relative centrifugal force (rcf) of 13,000 for 10 min. The aqueous phase was mixed with 200 μl of 100% ethanol, and the mixture was transferred to Purelink RNA Mini columns (Thermo Fisher Scientific) for purification following the manufacturer’s instructions. RNA was treated with Turbo DNase (Thermo Fisher Scientific) according to the manufacturer’s instructions and stored at –80ºC. RNA-seq libraries were prepared following the Ovation Complete Prokaryotic RNA-Seq (NuGEN) kit instructions. The O395-N1, C6706, and Haiti H1 reads were aligned using the Geneious Prime (54), version 2019.0.4, software package’s default settings to the V. cholerae O395-N1 (NC_009456.1, NC_009457.1), V. cholerae O1 biovar El Tor strain N16961 (NC_002505.1, NC_002506.1), and V. cholerae Haiti H1 (NZ_CP006947.1, NZ_CP006948.1) reference genomes, respectively. Differential expression analysis was performed using the DESeq2 (33) R package within the Geneious interface. Sequencing statistics are listed in Table S1.

### qRT-PCR.

Cells were harvested in mid-log phase, resuspended in 1 ml of TRIzol (Invitrogen), and incubated for 5 min at room temperature before the addition of 200 μl of chloroform. Samples were centrifuged at 13,000 relative centrifugal force (rcf) for 10 min. The aqueous phase was mixed with 200 μl of 100% ethanol, and the mixture was transferred to Purelink RNA Mini columns (Invitrogen) for purification following the manufacturer’s instructions. RNA was treated with Turbo DNase (Invitrogen) according to the manufacturer’s instructions. The KAPA SYBER FAST One-Step quantitative reverse transcription-PCR (qRT-PCR) kit (Kapa Biosystems) was used to measure mRNA abundance on the Eppendorf Mastercycler RealPlex2 system. Relative gene expression changes were calculated using the Livak method (55). The primers used for the detection of *hcp-1* and *ctxA* are listed in Table S5. The reference gene used was *gyrA*.

### Reciprocal best hits and secondary-structure prediction analysis.

To create a list of orthologous genes between the three strains, we used blastn to first find orthologs between N16961 and the other two strains (34). To confirm the identity of the orthologs obtained, the list of blastn hits was blasted back to N16961. Secondary-structure prediction was performed using the Phyre server (42). Protein structure models were analyzed using PyMOL 1.7.0.5 (Schrodinger, LLC).

### Data availability.

The data sets obtained in this study have been deposited in the Sequence Read Archive (SRA) under accession number PRJNA670462.
